# Obituary: Hidesaburo Hanafusa 1929–2009

**DOI:** 10.1186/1478-811X-7-7

**Published:** 2009-04-20

**Authors:** Stephan M Feller

**Affiliations:** 1 Weatherall Institute of Molecular Medicine, Oxford, UK

Hidesaburo Hanafusa, an eminent figure in the areas of virology, cancer signalling and protein interaction module research passed away at age 79 on 15 March 2009. His pioneering research provided the groundwork for the discovery of the first oncogene and also introduced protein – protein interactions as a novel concept in oncogenic signalling.

'Saburo' [meaning 'third-born son'], as most people knew him, was an inspiring scientist and a much beloved mentor to a large number of scientist currently conducting ground breaking research around the world. Modestly omitting the glorifying portion of his first name 'Hide-', i.e. 'excellent', was a clear tell-tale-sign of his personality.

Saburo always presented himself very calmly and gentlemanly, even shy, and he was never aggressive in promoting his outstanding research contributions, a somewhat rare trait in this day and age. For this he was greatly admired by many colleagues and his soft-spoken and well-chosen words carried much gravitas. As such, he was a vital figure in steering academic life at the renowned Rockefeller University in New York City, where he spent most of his scientific career.

Born on 1 December 1929 in Nishinomiya, Japan, Saburo received his bachelor degree in 1953, and his doctorate in 1960, both from Osaka University. In 1958, he married Teruko Inoue, a fellow student, who would also become an important and lifelong scientific colleague with whom he published over 35 papers between 1959 and 1992.

In 1961 Saburo accepted a postdoctoral position in the US, joining the laboratory of Harry Rubin, a pioneer in tumor virus research at Berkeley (University of California). This is where he began to work on the Rous Sarcoma Virus (RSV), the focus of his research for decades to come and one of the areas where his seminal findings gained him worldwide recognition and in 1982 the Lasker Award, often called the 'American Nobel Price'. In 1985 he was elected into the US National Academy of Sciences.

Saburo also received many other awards, including the Alfred P. Sloan, Jr. Prize in 1993 and the highly prestigious 'Bunka Kunsho', Japan's Order of Culture Award, presented to him by the Japanese Emperor, in 1995 and an honorary doctoral degree from Rockefeller University in June 2000.

Following his first research successes with RSV in the Rubin laboratory, Saburo moved to Paris in 1964, where he worked as a visiting scientist at the College de France until 1966. Returning to the USA, albeit the east coast, in 1966 he was appointed as head of the viral oncology laboratory at the Public Health Research Institute in New York City. In 1973, Saburo became professor of viral oncology at the Rockefeller University, the place where Peyton Rous had first isolated RSV (CT1) from a chicken tumor in 1911. He held this post at Rockefeller for 25 years, formally retiring from it in October 1998 at the age of 69.

Apart from his many groundbreaking findings with RSV and its oncogene, v-Src, his research at Rockefeller on adaptor proteins revolutionized the field of cell signalling. Bruce Meyer in his group isolated and characterised a new oncogene v-Crk, from the CT10 virus, which had been collected in 1931 but remained virtually unstudied for over half a century. This led to the discovery of Src Homology 2 and 3 (SH2 and SH3) domains as protein interaction modules in normal and oncogenic signal transmission.

Saburo's laboratory at Rockefeller was particularly attractive to graduate students and he helped to start many productive careers by carefully listening, providing valuable advice and, where needed, gently steering the novices through their first research steps. He also promoted several talented postdocs in his group to assistant professor, and, by allowing them to remain in his group for a few more years, enabled them to benefit from the inspiring research environment and state-of-the art infrastructure at Rockefeller during their initial years as independent scientists.

Upon retiring from Rockefeller, Saburo moved back to his home country Japan, where he became the director of the Osaka Bioscience Institute (OBI). Some of his former students and postdocs joined him at the OBI and continued to flourish under his guidance.

From the year 2000 onwards, the OBI hosted several symposia of alumni and current members from Saburo's laboratories, which brought together researchers from around the globe. These gatherings had the distinctive feel of a family reunion, providing the opportunity to hear some world-class science while enjoying a few of the many highlights of Japanese culture and cuisine, not to mention the heart warming hospitality displayed by all involved in organising these much cherished reunions [see Additional file 1].

In 2005 Saburo had to step down prematurely from this director post, due to his failing health. Nevertheless he continued to have a research group at the OBI and he remained intimately involved in mentoring scientists at various stages of development until his death from liver cancer in March of this year.

Saburo will be missed very much.

Thankfully, with his inspiring example on how to succeed in style he has left an important legacy that young scientists can aspire to. It will echo in the scientific community, in the continuing Hanafusa Alumni Symposia and beyond, for a long time to come.

## Supplementary Material

Additional file 1 **Movie link to OBI Symposium 2004.** Click here for file

